# Decomposition of the decoupling of CO_2_ emissions from economic growth in Ghana

**DOI:** 10.1186/s43093-022-00138-4

**Published:** 2022-08-06

**Authors:** Eric Fosu Oteng-Abayie, Foster Awindolla Asaki, Maame Esi Eshun, Eric Abokyi

**Affiliations:** 1 Department of Economics, Kwame Nkrumah University of Science and Technology, Kumasi, Ghana; 2 Public Utilities Regulatory Commission (PURC), Accra, Ghana; 3 Department of Economics, Universita‘ Politecnica Delle Marche, Ancona, Italy

**Keywords:** CO_2_ emissions, Economic growth, Decoupling, Logarithmic mean Divisia index, Tapio elasticity

## Abstract

The study analysed the relationship between CO2 emissions and economic growth in Ghana, specifically by analysing Ghana's decoupling status from 1990 to 2018. The Tapio elasticity method and the logarithmic mean Divisia index decomposition technique were used in the study to find out what causes CO2 emissions in Ghana to change over time. The analysis revealed that CO2 emissions and economic growth have increased over the study period, with economic growth driven mostly by the services and industrial sectors in the last decade. The decoupling index analysis shows that weak decoupling status dominated the period 1990–2018, interspersed with strong decoupling and expansive negative decoupling status. Economic structure and energy intensity, instead, were found to promote the decoupling of CO2 emissions and economic growth. From the decomposition analysis, CO2 emissions in Ghana are driven on the average by economic activities, emission factors, and population growth. To achieve the Sustainable Development Goal 13, the study suggests that policies to cut CO2 emissions should focus on economic activities, factors that affect emissions, and population growth. Also, to decouple CO2 emissions from economic growth, the implementation of policies that change the structure of the economy and energy intensity towards renewable sources should be intensified in Ghana.

## Introduction

The importance of climate change as a global issue is highlighted by its negative impact on the economic system. As noted by the literature, global warming and climate change have the potential to make life and natural ecosystems extinct [1]. Yet, one of the key drivers of global warming and its corresponding climate change is the emission of greenhouse gases (GHG). A key component of GHG is carbon dioxide (CO_2_). Ren et al. [2] argue that CO_2_ emissions are rising, contributing to global warming and threatening energy security and climate change. Similarly, CO_2_ emissions, since the pre-industrial era in the eighteenth century, have risen exponentially from an average of 280 parts per million (ppm) to about 414.72 ppm in 2021 due largely to the conversion of fossil fuels to energy by humans [3]. The quantum of CO_2_ in the atmosphere is forecasted to increase to 530 ppm by 2050 and 780 ppm by 2100 if nothing is done [4].

The global priority, under the Paris COP21 agreement, is to reduce the average temperature levels emanating from the rising CO_2_ emissions. The main objective is to limit the rising global temperature to ${2}^{\circ }\mathrm{C}$ but has set a target of reducing it to ${1.5}^{\circ }\mathrm{C}$ [5]. According to the UNFCCC [5], limiting temperature rise to ${1.5}^{\circ }\mathrm{C}$ requires a 45% reduction in annual CO_2_ emissions by 2030 and a net-zero reduction by 2050. This is a big challenge because the Global Carbon Update 2021 reports that carbon dioxide emissions released from fossil fuel consumption rise every ten years. For instance, the yearly average emissions rose to 35 tons of carbon dioxide in the 2010s from 11 billion tons of carbon dioxide a year in the 1960s [6].

Recently, the COVID-19 pandemic caused a reduction in CO_2_ emissions worldwide as a result of restrictions on both economic and industrial activities [7]. Evidence suggests that CO_2_ emissions decreased by almost 7% on average in 2020 compared to 2019, with the first half of 2020 seeing the highest reduction of 8.8% ([6] and International Energy Agency 2020). For developing nations, a 5% decrease on average in CO_2_ emissions was recorded in 2020 due to the COVID-19 pandemic [8, 9]. In Ghana for instance, CO_2_ emissions decreased from 17.7 million tonnes in 2019 to 16.5 million tonnes in 2020, a decline of 6.85% [10]. The reduction in CO_2_ emissions globally may not be sustainable in the long run, especially as global economic activities slowly return to pre-COVID-19 state (Ray et al. 2022). Indeed, CO_2_ emissions rebounded in 2021 to pre-pandemic levels in 2019 [6].

The empirical literature on Ghana has identified a positive relationship between CO_2_ emissions and economic growth [11]. The implication is that CO_2_ emission rise with any marginal increase in economic growth. This positive relationship is also asserted to be the result of population growth, energy consumption, and the structure of the economy [11]. Admittedly, Cederborg and Snöbohm [12] among others affirm that economic growth rises with CO_2_ emissions but lacks a turning point where CO_2_ emissions decline with higher economic growth.

A number of CO2 emissions policies and programmes have been implemented in Ghana with the objective of mitigating CO_2_ emissions. Among these are fuel diversification for thermal electricity, installation of power factor correction devices, solar lantern replacement programme, sustainable land and water management projects, and forest investment programmes [13]. Despite efforts to reduce CO_2_ emissions in Ghana, the data reveal that CO_2_ emissions are still rising [10], albeit in recent times, the COVID-19 pandemic has contributed to a decline in CO_2_ emissions in developing countries [8, 9], and [7].

Several empirical studies have used time-series econometric approaches to investigate Ghana's the connection between CO_2_ emissions and economic growth. For example, Osadume and University [11], Abokyi et al. [14], Appiah [15], and Asumadu-Sarkodie and Owusu [16] address the links and causality between CO_2_ and economic growth and other variables such as energy use, population expansion, and industrial growth. Notwithstanding, the emphasis on CO_2_ emissions and economic growth, prior studies have overlooked the underlying sectoral and structural causes of CO_2_ emissions, particularly in Ghana. It should be noted that efforts to reduce CO_2_ emissions are likely to be ineffective unless the underlying sources of emissions are identified and targeted. This study seeks to fill this gap.

Moreover, although the logarithmic mean Divisia index (LMDI) and Tapio elasticity methods have been used in several studies in various sectors of the global economy [2, 17–20], no known empirical studies on CO_2_ emissions and economic growth relationships in Ghana using these methods have been published. This study will employ both the decomposition and decoupling methods to fill this gap.

Focusing on Ghana’s environmental footprints from 1990 to 2018, the findings will inform future predictions and policy recommendations for reducing GHG emissions while encouraging economic growth. In this regard, the study decouples CO_2_ emissions from economic growth and investigates Ghana's decoupling status. Findings from this analysis could point out the key factors that tie CO_2_ emissions to economic growth and help policymakers design policies towards achieving sustainable economic growth and the Sustainable Development Goals 13. Furthermore, the study disaggregated CO_2_ emissions into various categories and investigated the drivers of CO_2_ emissions in Ghana. This analysis therefore has strong policy relevance.

The next section reviews the existing theoretical and empirical literature related to the study. The methodology, analytical techniques, and data sources are described in section "Methods and data". The discussions and presentation of results as well as the policy implications of the study results are highlighted in sections "Results and discussion" and "Conclusion and policy implications" accordingly.

## Definitions and types of decoupling concepts

Decoupling, a measure of successful economic and environmental integration [21], has recently gained traction in the literature on CO_2_ emissions and economic growth relationships. The OECD [22] explains decoupling as removing the connection between economic “bads” and economic goods. This entails addressing or resolving environmental challenges without jeopardising economic growth. Decoupling happens when emission growth rates are steady or lower than an economy's growth pace.

The two primary forms of decoupling are absolute decoupling and relative decoupling. The state where environmental pressures are identified to be reducing or at best stable while the economy grows is termed as absolute decoupling. It involves the absolute or total reduction in CO_2_ emissions as economic activities expand. Absolute decoupling happens, according to Luken and Piras [23], as the rate of growth in energy demand is less than or equal to zero (0). When the growth rate of energy demand is zero or negative while the growth rate of the economy is positive. When economic growth rates exceed the pace of change in CO_2_ emissions, relative decoupling occurs. This is backed by empirical evidence [17, 22], which indicates that both economic and CO_2_ growth rates are on the rise, although CO_2_ growth is significantly slower than economic growth.

Tapio [24] reclassified decoupling criteria as coupling, decoupling, or negative decoupling. These are further classified into eight logical alternatives, which include strong decoupling, weak decoupling, expansive negative decoupling, strong negative decoupling, expansive coupling, recessive coupling, and expansive decoupling. Thus, Tapio's definition is more condensed than the OECD definition, as it incorporates additional environmental and economic growth aspects. The Tapio’s reclassifications are defined in the methodology section and used for further analysis in this study.

### Theoretical and empirical literature review

#### Theoretical literature review

The Environmental Kuznets Curve (EKC) hypothesis is one of the widely used theoretical prepositions used to understand the interlinkages between CO2 emissions and economic growth. The hypothesis posits that environmental pollution is nonlinearly correlated with economic growth ([25]; and Al 2007). In its beginning phase, economic growth often leads to greater pollution due to intense resource use. With a robust economic structure and the accumulation of energy-saving technology, economic expansion can reduce resource consumption and pollution [26]. Engo [17] termed the process as decoupling. Zhang [27] introduced decoupling analysis in the early 2000s to examine the connection between CO_2_ emissions and economic growth. Decoupling was later characterised as an indicator by the OECD [28]. However, different metrics and methods of analysis, such as econometric analysis, OECD decoupling analysis, IGTX decoupling method, variation analysis method, and the Tapio elasticity method, can be used in decoupling analysis. Zhong et al. [29, 30] posited that there is no better method of decoupling. The Tapio elasticity method is, however, the most extensively utilised and agreed-upon method of analysis by researchers in studying the relationship between economic growth and environmental problems. One advantage of the Tapio elasticity method is that it has developed eight logical possibilities necessary for determining decoupling statuses.

One drawback of the Tapio method is that it does not expound on the fundamental reason for the decoupling state. To overcome this shortfall, decoupling indicators are classified into separate elements using Zhang et al.’s (2015) LMDI method. Improvements to the Divisia index methods by Ang and Lee [31], Liu et al. (1992), and Ang and Choi [32] have given the LMDI more theoretical and practical advantages, making it a widely used analytical technique by researchers among the several index decomposition analysis methods such as Laspeyres, Paasche, and Marshall–Edgeworth indices. Thus, LMDI provides flawless decomposition and can be used on more than two elements. Furthermore, when aggregated, one is likely to obtain consistent estimates between subgroup level and overall group level results (Ang and Liu 2001; [33]). Notably, global efforts to reduce GHG emissions have led to the wider application of the LMDI technique to determine the components that drive carbon dioxide (CO_2_) emissions. Many studies have applied LMDI decomposition analyses to energy studies [34–37] and CO_2_ emissions analyses [38, 39]. Recent literature has focused on CO_2_ emissions and economic growth on specific sectors Wang and Wang [7] and CO_2_ emissions and renewable energy [40] and [8, 9]. However, no study has considered decomposition of economic growth and CO_2_ emissions in the context of Ghana. Therefore, it is essential to decompose economic growth and identify the factors that significantly affect growth of CO_2_ emissions in Ghana so as to target such factors to reduce overall CO_2_ emissions and its adverse impact on the sustainability of the environment.

#### Empirical literature review

Empirically, several studies have employed the Tapio elasticity method in decoupling analysis. For example, Dong et al. [41] investigated economic growth and energy use in Liaoning Province. The findings revealed four decoupling states: expansive coupling, expansive negative decoupling, weak decoupling, and strong decoupling. Similarly, Wu et al. [26] assessed the state of decoupling between CO_2_ emissions and economic development in both poor and affluent countries. The findings suggested that affluent countries had a robust decoupling state, whereas poor countries had weak and variable decoupling states with no regularity. In Taiwan, the decoupling situation between industrial growth and CO_2_ emissions was also explored using data from 2007 to 2013 [42]. The results revealed a negative decoupling in Taiwan's economy.

While some studies used the Tapio elasticity method exclusively, other studies also applied the LMDI method of analysis. Wang and Feng [43], for example, scrutinised the effects of economic development, emission factor, population, energy structure, industrial structure, and energy intensity on CO_2_ emissions in China between 2000 and 2014. According to the findings, economic expansion increases CO_2_ emissions, whereas energy intensity decreases CO_2_ emissions. In the same way, Li et al. (2017) studied the factors that drive the emission of CO_2_ in 11 countries that contribute 67% of global warming with data spanning from 1990 to 2013. The study found that emission factors, population, and economic activity tended to increase CO_2_ emissions, whereas the share of electricity generation, energy intensity, share of thermal energy production, and electricity intensity appeared to decrease CO_2_ emissions.

Other research also analysed data using both the Tapio and LMDI techniques. Engo [17] studied the relationship between CO_2_ emissions and economic growth in Cameroon using data from 1990 to 2015. The results revealed three levels of decoupling: weak negative decoupling, strong negative decoupling, and strong decoupling. The findings also suggested that energy intensity, demographic shifts, and economic activity were factors that favoured negative decoupling, whereas economic structure and emission factors favoured strong decoupling. Hossain and Chen [18] employed the same methodology and discovered that Bangladesh attained weak decoupling all throughout the analysis periods, with the exception of the final period (2015–2017), where significant decoupling was attained. They also discovered that a change in scale effect causes a considerable increase in CO_2_ emissions and economic structure, despite the fact that energy intensity has a minimal influence on the growth in CO_2_ emissions.

A paucity of empirical literature exists in the context of countries in Sub-Saharan Africa. None of the existing treatise, except Tenaw [20] in Ethiopia, evaluated the decoupling status of countries in Sub-Saharan Africa, independently. To the best of the researchers’ knowledge, this is the first decoupling and decomposition treatise using both the Tapio elasticity and the LMDI methods to investigate the relationship between CO_2_ emissions and economic growth in Ghana.

## Methods and data

### Analytical methods and model specifications

In the empirical literature, several methods of analysis or estimation techniques such as regression analysis, STIRPAT model, panel cointegration, LMDI decomposition, Tapio elasticity method, ARDL model, and many estimation techniques have been employed in environmental pressure analysis [44, 45].

For three reasons, we use both the Tapio elasticity and the LMDI methods of analysis, as recommended by Engo [17]. First, they are widely used by most researchers whose research objectives include surveying the connection between environmental concerns and economic growth [17]. Second, the Tapio elasticity estimate technique is employed specifically to detect whether a state is decoupling or not. Third, the LMDI method decomposes the decoupling indicators into different factors and examines the various factors that account for or drive the emission of CO_2_ in an economy. Applying both methods in the case of Ghana will help reveal the trends, the state of decoupling, and the factors that drive the emissions of CO_2_ and guide policy in CO_2_ emissions mitigation. This approach was first introduced in the IPCC in the 1990s by Kaya [46], and it expresses CO_2_ emissions as four identities (factors) as shown in Eq. (1). However, in this study, the Kaya model Eq. (1) is extended to include energy intensity per unit of GDP as expressed in Eq. (2).

#### LMDI decomposition

As shown in Eq. (1), we employed the Kaya [46] model, which expresses CO_2_ emissions in terms of four major factors: CO_2_ emissions (C), energy consumption (E), economic activity (GDP), and population growth (P) as

1 $$ C = \frac{C}{E} \times \frac{E}{GDP} \times \frac{GDP}{P} \times P. $$

To ascertain the changes in CO_2_ emissions emanating from energy consumption in the economy of Ghana, the extended Kaya Identity, which is defined as the intensity of energy consumption per unit of GDP per capita, is given in Eq. (2) as

2 $$ C = \frac{{C_{i} }}{{E_{i} }} \times \frac{{E_{i} }}{{GDP_{i} }} \times \frac{{GDP_{i} }}{GDP} \times \frac{GDP}{P} \times P $$

where *C* represents total emission of CO_2_, $$C_{i}$$, $$E_{i}$$, and $$GDP_{i} $$ denotes CO_2_ emissions from sector *i*, energy consumption in sector *i*, and economic output in sector *i*, respectively. Equation (2) can also be expressed as:

3 $$ C = f \times I \times ES \times EA \times P $$

where $f = \frac{{C_{i} }}{{E_{i} }}$ is the emissions factor,^1^$I = \frac{{E_{i} }}{{GDP_{i} }}$ represents the energy intensity of sector *I*, $ES = \frac{{GDP_{i} }}{GDP}$ represents the share of economic output in sector *i* (economic structure), $EA = \frac{GDP}{P}$ represents economic activities in the economy.

LMDI is defined by Ang (2015) as a change in CO_2_ emissions from the base year to the target year. The LMDI technique of total CO_2_ emissions can be stated using the additive approach as follows from Eqs. (4) to (9).

4 $$ \Delta C_{TOT} = C^{t} - C^{0} = \Delta C_{f} + \Delta C_{I} + \Delta C_{ES} + \Delta C_{EA} + \Delta C_{P} $$

where

5 $$ \Delta C_{f} = \mathop \sum \limits_{i} \frac{{C_{i}^{t} - C_{i}^{0} }}{{lnC_{i}^{t} - lnC_{i}^{0} }} \times \ln \left( {\frac{{f^{t} }}{{f^{0} }}} \right) $$

6 $$ \Delta C_{I} = \mathop \sum \limits_{i} \frac{{C_{i}^{t} - C_{i}^{0} }}{{lnC_{i}^{t} - lnC_{i}^{0} }} \times \ln \left( {\frac{{I^{t} }}{{I^{0} }}} \right) $$

7 $$ \Delta C_{ES} = \mathop \sum \limits_{i} \frac{{C_{i}^{t} - C_{i}^{0} }}{{lnC_{i}^{t} - lnC_{i}^{0} }} \times \ln \left( {\frac{{ES^{t} }}{{ES^{0} }}} \right) $$

8 $$ \Delta C_{EA} = \mathop \sum \limits_{i} \frac{{C_{i}^{t} - C_{i}^{0} }}{{lnC_{i}^{t} - lnC_{i}^{0} }} \times \ln \left( {\frac{{EA^{t} }}{{EA^{0} }}} \right) $$

9 $$ \Delta C_{P} = \mathop \sum \limits_{i} \frac{{C_{i}^{t} - C_{i}^{0} }}{{lnC_{i}^{t} - lnC_{i}^{0} }} \times \ln \left( {\frac{{P^{t} }}{{P^{0} }}} \right). $$

Equation (4) shows the total changes of CO_2_ emissions $\left( {\Delta C_{TOT} } \right)$ during the study period are affected by the sum of the changes of CO_2_ emissions factor at the sector level $\left( {\Delta C_{f} } \right)$, changes of energy consumption intensity at the sector level $\left( {\Delta C_{I} } \right)$, structural changes in economic activity $\left( {\Delta C_{ES} } \right)$, total changes in economic activity $\left( {\Delta C_{EA} } \right)$, and changes in population size and lifestyle $\left( {\Delta C_{P} } \right)$, respectively.

#### Tapio (elasticity) decoupling index

Following Tapio [24], the decoupling index of energy-related CO_2_ emissions from economic growth between the base year and the target year can be expressed as Eq. (10).

10 $$ D_{C, G} = \frac{\beta C}{{\beta G}} = \frac{{\frac{{C^{t} - C^{0} }}{{C^{0} }}}}{{\frac{{G^{t} - G^{0} }}{{G^{0} }}}} = \frac{{\Delta C \times G^{0} }}{{C^{0} \times \Delta G}}. $$

Equation (10) can also be respecified as Eq. (11).

11 $$ D_{C, G} = \Delta C \times \frac{{G^{0} }}{{C^{0} \times \Delta G}}. $$

Here, $$D_{C, G}$$ is the decoupling index, $$C^{t}$$ and $$C^{0}$$ are the current and previous CO_2_ emissions levels, and $$G^{t} $$ and $$G^{0}$$ are current and previous economic growth rates. Also, $$\Delta G$$ and $$\Delta C$$ represent changes in economic growth and CO_2_ emissions, respectively. Furthermore, $$\beta C = \frac{{C^{t} - C^{0} }}{{C^{0} }}$$ and $$\beta G = \frac{{G^{t} - G^{0} }}{{G^{0} }}$$ are defined as the rate of growth of CO_2_ and economic growth between the base and current year. The decoupling index ($$D_{C, G}$$) is obtained by combining Eqs. (4) and (11) resulting in Eq. (12).

12 $$ D_{C, G} = \Delta C_{TOT} \times \frac{{G^{0} }}{{C^{0} \times \Delta G}}. $$

Equation 12 can further be expressed as

13 $$ D_{C, G} = (\Delta C_{f} + \Delta C_{I} + \Delta C_{ES} + \Delta C_{EA} + \Delta C_{P} ) \times \frac{{G^{0} }}{{C^{0} \times \Delta G}} $$

14 $$ D_{C, G} = D_{f} + D_{I} + D_{ES} + D_{EA} + D_{P} $$

15 $$ D_{f} = \left[ { \mathop \sum \limits_{i} \frac{{C_{i}^{t} - C_{i}^{0} }}{{lnC_{i}^{t} - lnC_{i}^{0} }} \times \ln \left( {\frac{{f^{t} }}{{f^{0} }}} \right)} \right] \times \frac{{G^{0} }}{{C^{0} \times \Delta G}} $$

16 $$ D_{I} = \left[ { \mathop \sum \limits_{i} \frac{{C_{i}^{t} - C_{i}^{0} }}{{lnC_{i}^{t} - lnC_{i}^{0} }} \times \ln \left( {\frac{{I^{t} }}{{I^{0} }}} \right)} \right] \times \frac{{G^{0} }}{{C^{0} \times \Delta G}} $$

17 $$ D_{ES} = \left[ { \mathop \sum \limits_{i} \frac{{C_{i}^{t} - C_{i}^{0} }}{{lnC_{i}^{t} - lnC_{i}^{0} }} \times \ln \left( {\frac{{ES^{t} }}{{ES^{0} }}} \right) } \right] \times \frac{{G^{0} }}{{C^{0} \times \Delta G}} $$

18 $$ D_{EA} = \left[ { \mathop \sum \limits_{i} \frac{{C_{i}^{t} - C_{i}^{0} }}{{lnC_{i}^{t} - lnC_{i}^{0} }} \times \ln \left( {\frac{{EA^{t} }}{{EA^{0} }}} \right)} \right] \times \frac{{G^{0} }}{{C^{0} \times \Delta G}} $$

19 $$ D_{P} = \left[ { \mathop \sum \limits_{i} \frac{{C_{i}^{t} - C_{i}^{0} }}{{lnC_{i}^{t} - lnC_{i}^{0} }} \times \ln \left( {\frac{{P^{t} }}{{P^{0} }}} \right)} \right] \times \frac{{G^{0} }}{{C^{0} \times \Delta G}}. $$

The total decoupling index of CO_2_ emissions through energy-related factors and economic growth is denoted by $D_{C, G}$ and $D_{f} ,D_{I} , D_{ES} , D_{EA} , D_{P}$ represents the decoupling indices of emissions factor, economic activity, energy intensity, population size, and economic structure. Following Tapio [24], the decoupling statuses of these indices are determined based on the eight classes of decoupling. The criteria for the classifications are provided in Table 1.

**Table 1:** Criterion for defining the decoupling status

Decoupling status	${D}_{C, G}$	$\beta C$	$\beta G$
1 Strong decoupling	${D}_{C, G}$<0	< 0	> 0
2 Weak decoupling	0 $.8 \ge {D}_{C, G}>0$	> 0	> 0
3 Expansive decoupling	1.2 ≥ ${D}_{C, G}$> 0.8	> 0	> 0
4 Expansive negative decoupling	${D}_{C, G}$> 1.2	> 0	> 0
5 Strong negative decoupling	${D}_{C, G}$< 0	> 0	< 0
6 Weak negative decoupling	0.8 ≥ ${D}_{C, G}$> 0	< 0	< 0
7 Recessive coupling	1.2 ≥ ${D}_{C, G}$> 0.8	< 0	< 0
8 Recessive decoupling	${D}_{C, G}$> 1.2	< 0	< 0

*Source*: Tapio [24]

### Data description

Data for the study span 1990 to 2018 and were extracted from the World Development Indicators (WDI) [10]. The study period was influenced by the availability of data and complete information on the variables employed in the investigation. Details of the variables used are described in Table 2.

**Table 2:** Data description

Variable	Definition	Measurement
$$GDP$$	Gross domestic product converted to growth rate	GDP in billions (LCU)
$${CO}_{2}$$	Total carbon emission	CO_2_ emissions (kt)
$$GDPE$$	GDP per energy consumption	GDP per unit of energy use (PPP $ per kg of oil equivalent)
$$P$$	Total population	The total population includes all residents in Ghana
$$E$$	Energy consumption	Energy use (kg of oil equivalent per capita)
$${C}_{i}$$	carbon emission from sector $$i$$,	CO_2_ emissions from the energy sector

*Source*: WDI 2020

### Descriptive summary of variables

Table 3 presents a summary statistic of the variables described in Table 2. The average GDP over the study period was GHC53.6 billion. However, this was coupled with an average CO2 emission of 8281.72 kt and an average GDP of the energy sector of $8.45 per kg of oil equivalent. Moreover, the average energy consumption over the study period was 329.92 kg of oil equivalent per capita, with a corresponding CO2 emission of 7649.36 tCO_2_ in the energy sector. The average population during the study period was 21.7 million.

**Table 3:** Descriptive summary of variables

Variables	Mean	SD	Min	Max
$GDP$	53,600	84.800	192.00	300,000
$CO2$	8381.72	4420.66	2560.00	16,110.00
$GDPE$	8.45	4.62	3.45	16.26
$E$	329.92	41.97	266.12	408.25
$Ci$	7649.36	3441.04	3237.96	15,368.40
$P$	21.70	4.62	14.8	29.80

Source: Authors' construct

## Results and discussion

### Trends of CO_2_ emissions and economic growth

**Fig. 1:**
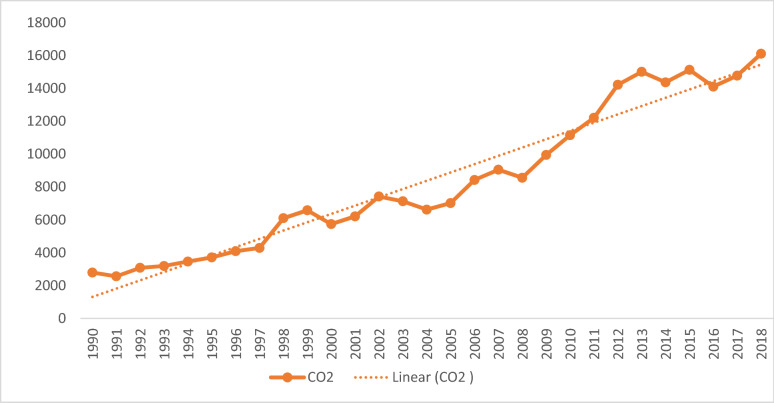
Trend of CO_2_ emissions in Ghana. Source: Authors' construct

Figure 1 shows the trends of CO_2_ emissions in Ghana from 1990 to 2018. From Fig. 1, CO_2_ emissions showed a steady rise from 1990 to 1997 but exhibited some fluctuations from 1998 to 2008, although the overall trend was positive. Beyond 2008, CO_2_ emissions further showed an upward trend until 2013, after which it began to fluctuate again, but resumed the upward trend after 2016. The fluctuations in the growth of CO_2_ emissions may be attributed to the efforts made by the Government of Ghana to mitigate the emission of CO_2_. Twerefou et al. [47] showed an increase in CO_2_ emissions in Ghana from 12.2 to 23.9 Mt between 2000 and 2010, respectively. This, however, confirms the result that CO_2_ is rising and remains a major concern in Ghana.

**Fig. 2:**
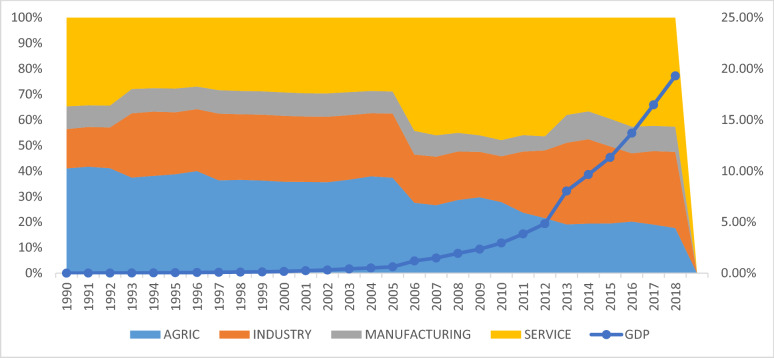
Trends of economic growth in Ghana. Source: Authors' construct

Figure 2, which displays the trends in economic growth in Ghana, also depicts an upward trend, suggesting that generally, GDP rises with CO_2_ emissions in Ghana. For the period from 1990 to 2005, economic growth was positive, although the rate was negligible. A steady rise in economic growth was witnessed from 2006 to 2012, and a steeper rise afterwards. As with all economies, Ghana's economic growth is determined by three sectors: agriculture, industrial, and the service or tertiary sector. It can be observed that economic growth in Ghana from 1990 to 2005 was largely contributed to by the agricultural sector. The industrial sector's contribution to economic growth increased after 1992 but dropped in the 2006 to 2010 period. Meanwhile, the service sector has been the most important sector in terms of its contribution to GDP since 2006. The sharp rise in economic growth recorded from 2012 onwards can be attributed to the industrial sector’s output growth. This result is supported by GSS (2018) findings which postulate that the industrial sector, including the manufacturing sector, in Ghana accounts for 23.68% of the growth of the Ghanaian economy. According to Fig. 2, the services sector accounted mainly for the recent economic growth. This confirms the findings of O'Neil [48] that posited that the contributions of the industrial sector, agricultural sector, and the service sector are 29.74%, 19.25%, and 45.01%, respectively, to economic growth. The service sector, based on the Perez-Lopez [49] classification, includes transport, repair of vehicles, household goods, storage, wholesale and retail trade, communications, finance, insurance, real estate, restaurants and hotels, and business services. These activities are the core economic activities that contribute most to Ghana's economic prosperity. Notably, a report published by GSS [50] ascertained that the economic growth rate in Ghana for 2021 was 5.4%, with the service sector, agricultural sector, industry sector, and manufacturing sector contributing 9.4%, 8.4%, 0.8%, and 7.8%, respectively, to economic growth, thus clearly confirming that economic growth in Ghana has recently been driven by the service sector, as the trend shows in Fig. 2.

Both CO_2_ emissions and economic growth over the years have been increasing. However, the recent rise in CO_2_ emissions in Ghana can be associated with the tertiary or service sector as the leading sector contributing more to CO_2_ emissions in Ghana, although the industrial sector (comprising manufacturing, lumbering, mining, food processing, aluminium smelting, cement, small commercial shipbuilding, and petroleum industries), has played a significant role in the last decade. From a contextual point of view, transport services, energy-related services, waste accruing from human activities, and open burning of waste increased the contribution of CO_2_ emissions by 45.8%, 22.1%, 14.4%, and 92.5% between 2016 and 2019, respectively (Ghana’s Third Biennial Climate Update report, [51]. This, however, is an indication of recent CO_2_ emissions being driven by the service sector activities in Ghana. This result supports the empirical findings of Appiah [15], which posits that economic growth and CO_2_ emissions are positively correlated in Ghana.

### Analysis of decoupling status of CO_2_ emissions from economic growth

The analysis of the decoupling status of CO2 emissions from economic growth is presented in Table 4. Over the period 1990 to 2018, there was weak decoupling of CO2 emissions from economic growth. The cumulative average decoupling index for the period was 0.2548, with CO2 growth and economic growth rates of 7.01% and 30.89%, respectively. This means during the period, Ghana experienced growth in CO2 emissions with economic growth. The low emissions of CO2 can be ascribed to implementation of the carbon mitigation policies [13] and low levels of emission factors reported in Ghana compared to advanced countries. From Fig. 2, the economy of Ghana has been mainly driven by the service and the agriculture sectors in terms of contributions to GDP, until the last decade where the industrial sector growth experienced tremendous growth from 2005 to 2018. The overall weak decupling is not surprising, given the primary nature of production and exports of goods in Ghana, which has low CO2 emissions content. According to the CAIT Climate Watch [52], between 1990 and 2018, CO_2_ emissions have been largely influenced by growth in emissions from electricity and heat generation to the tune of 7250%, followed by manufacturing and construction with a growth rate of 670%. Transportation and industry sectors follow with 397% and 267% growth rates, in relative terms. These are sectors that have been important to Ghana’s growth path. Specifically, the fast growth of the services sector, especially trading in finished goods, has very low emission factors and contributes to slow growth in CO_2_ emissions while economic growth is rising. In the energy sector, Ghana until the last decade had historically depended on hydropower with low emissions in its energy-mix to drive its economic growth. Currently, there is high resort to thermal energy sources with relatively high emissions factors and coupled with the industrialisation drive that depends heavily on fossil fuels and gas, decoupling CO_2_ emissions from economic growth might be a challenge in Ghana. This finding is supported by existing studies that found that most developing countries have not been successful in decoupling CO2 emissions from economic growth [53].

**Table 4:** Decoupling of CO_2_ emissions from economic development (1990–2018)

Year	${D}_{C, G}$	$\beta C$	$\beta G$	Decoupling state
1990–1991	−0.3104	−0.0824	0.2656	Strong
1991–1992	1.3340	0.2031	0.1523	Expansive negative
1992–1993	0.0935	0.0357	0.3821	Weak
1993–1994	0.2444	0.0846	0.3463	Weak
1994–1995	0.1482	0.0723	0.4875	Weak
1995–1996	0.2236	0.1024	0.4581	Weak
1996–1997	0.1973	0.0489	0.2478	Weak
1997–1998	1.8590	0.4219	0.2270	Expansive negative
1998–1999	0.4125	0.0787	0.1908	Weak
1999–2000	−0.3985	−0.1277	0.3204	Strong
2000–2001	0.2043	0.0819	0.4007	Weak
2001–2002	0.6874	0.1948	0.2835	Weak
2002–2003	−0.1105	−0.0391	0.3538	Strong
2003–2004	−0.3456	−0.0715	0.2069	Strong
2004–2005	0.2775	0.0604	0.2178	Weak
2005–2006	0.2179	0.2009	0.9219	Weak
2006–2007	0.3056	0.0735	0.2406	Weak
2007–2008	−0.1794	−0.0541	0.3017	Strong
2008–2009	0.7662	0.1624	0.2119	Weak
2009–2010	0.4696	0.1206	0.2568	Weak
2010–2011	0.3169	0.0951	0.3000	Weak
2011–2012	0.6351	0.1646	0.2592	Weak
2012–2013	0.0842	0.0556	0.6600	Weak
2013–2014	−0.2132	−0.0426	0.2000	Strong
2014–2015	0.3051	0.0529	0.1733	Weak
2015–2016	−0.3207	−0.0674	0.2102	Strong
2016–2017	0.2352	0.0475	0.2019	Weak
2017–2018	−0.0047	0.0900	0.1719	Weak
1990–2018	0.2548	0.0701	0.3089	Weak

*Source*: Authors' construct ${\mathrm{D}}_{\mathrm{C},\mathrm{ G}},\mathrm{ \beta C},\mathrm{ and \beta G}$ denote decoupling index, the rate of growth of CO_2_, and economic growth, respectively

From Table 4, Ghana’s weak decoupling state was interspersed with strong and expansive negative decoupling states during the period. Strong decoupling states occurred in 1990–1991, 1999–2000, 2002–2004, 2007–2008, 2013–2014, and 2015–2016. Expansive negative decoupling occurred in 1990–1991 and 1997–1998. The strong decoupling status is viewed as the best status for CO_2_ reduction, suggesting that the speed of economic growth has been faster than the rate of increase in CO_2_ emissions. This could also be attributed to the temporary environmental and CO_2_ mitigation strategies such as fuel diversification for thermal electricity, installation of power factor correction devices, solar lantern replacement programmes, sustainable land water management projects, and forest investment programmes [13].

### Decomposition of decoupling indicators

**Fig. 3:**
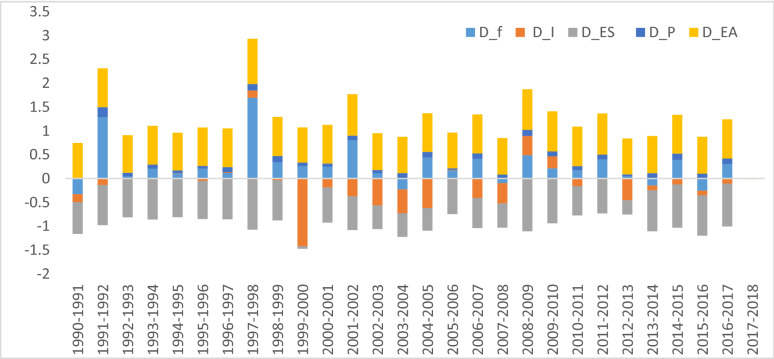
Decomposition of decoupling indicators. Source: Authors' construct

Figure 3 shows the result of the decomposition analysis of the decoupling indicators in Ghana from 1990 to 2018. The result indicates that changes in economic activities (DEA) and economic structure (DES) contributed significantly to the growth in CO2 emissions in Ghana in the periods of weak decoupling. On the other hand, growth in energy intensities (DI) and emissions factors (Df) reinforced economic activities (DEA) and economic structure (DES) to realise the strong and negative expansive decoupling status. This implies that improvement in production efficiency and the deployment of green energy technologies in Ghana will help in the absolute decoupling of economic growth from CO2 emissions in the long term. Environmental degradation is expected to decrease as a result of improved technology, increased environmental awareness, and the effective application of environmental regulations brought on by economic development. The result of this study supports the findings of [54, 55] that the main factor limiting CO2 emissions growth is energy consumption intensity in the strong decoupling periods. Also, in line with [54, 55], Ghana's economic structure and economic activities promote CO2 emissions, especially in the weak decoupling periods.

### Decomposition of annual changes in CO_2_ emissions in Ghana

From Table 5 (and Table 6 in Appendix), the analysis shows that changes in economic activities ($\Delta {\varvec{C}}_{{{\varvec{EA}}}}$) are the main driver of growth in CO_2_ emissions in Ghana, with 51,343.98 tCO_2_, corresponding to 15.31%. Economic activities are a key source of funds for a country's development, and thus the government pays attention to these activities in funding its short and long-term projects. From Fig. 2, growth in the service and industrial sectors, dominant economic activities, contributed mostly to the growth of the economy, at least since 2010. This result is consistent with the findings of Appiah et al. [15], which found a positive relationship between economic growth rate and CO_2_ emissions in Ghana. The strength of this finding is backed up by the analysis in Fig. 3, which showed that the growth in CO2 emissions was driven by economic growth in the different economic sectors.

**Table 5:** Decomposition of changes in CO_2_ emissions (in percentages)

Period	$\Delta {C}_{f}$	$\Delta {C}_{I}$	$\Delta {C}_{ES}$	$\Delta {C}_{EA}$	$\Delta {C}_{P}$	$\Delta {C}_{TOT}$
1990–1995	5.83	1.36	9.43	7.82	8.24	7.68
1996–2000	24.70	21.17	10.09	10.95	12.06	16.95
2001–2005	20.78	30.16	13.09	15.02	15.70	10.69
2006–2010	25.23	3.93	27.52	23.91	21.59	34.48
2011–2015	23.02	39.05	27.76	32.99	30.25	33.23
2016–2018	0.44	4.35	12.12	9.30	12.17	−3.02
1990–2018	3.71	−4.15	−12.87	15.31	1.58	3.57

*Source*: Authors' construct

${C}_{f, } {C}_{I}, {C}_{ES}, {C}_{EA, } {C}_{P}, \mathrm{and} {C}_{TOT}$ denote emission factor, energy intensity, economic structure, economic activity, population, and sum of sector CO_2_, respectively

Moving away from economic activity, the next significant driver of growth in CO_2_ emissions in Ghana is the emission factor, which emitted an average of 12,430.33 tCO_2_ over the period, constituting 3.71%. In line with expectations, the IPCC (2019) asserted that a higher emission factor is associated with increased CO_2_ emissions. Emission factors can be used in the conversion of land use (clearing of forest and grassland for crop production) to be significant in releasing more CO_2_ into the atmosphere. Due to population growth, estate developers clear more forests for infrastructural purposes and other human activities such as waste disposals, mining activities, and transportation services, which pollute the environment.

Also, demographic factors, which are changes in the population size and lifestyle, emitted an average CO_2_ of 5309.75, representing 1.58%. This result could be explained by the growth in population size in Ghana, which corresponds to a surge in demand for energy, occasioning a high build-up in CO_2_ emissions. For instance, the electrification rate in Ghana increased over the past decades by 85% (GLSS 2017). Because of this, more energy is being used, which is partly to blame for the rise in CO2 emissions.

In contrast to the above findings, structural variations in energy intensity and economic activity reduced CO_2_ emissions in Ghana by 43,172.95 tCO_2_ and 13,933.04 tCO_2_, constituting 4.15% and 12.87% between 1990 and 2018, respectively. This confirms previous studies by Liu et al. [56] which suggested changes in the structure of an economy have a significant impact on CO_2_ emissions. For instance, industrial restructuring and modernisation of energy infrastructure reduce CO_2_ emissions (Wang and Watson 2010). This result can be attributed to structural changes in energy consumption brought about by Ghana's carbon reduction plan.

## Conclusion and policy implications

The study has addressed the interlinkages between economic growth and the emission of CO_2_ in Ghana with data ranging from 1990 to 2018 using the Tapio elasticity and the LMDI methods for estimation. The main objectives were to scrutinise the trends of CO_2_ emissions and economic growth, decouple CO_2_ from economic growth, and examine the drivers of CO_2_ emissions in Ghana.

From the findings, it can be adduced that both economic growth and CO_2_ emissions have both increased over the study period. The recent drivers of economic growth were associated with services and the industrial sector, which made increasing contributions to economic growth. The decoupling index analysis shows that weak decoupling status dominated the period 1990–2018. Furthermore, economic activity and economic structure contributed to the weak decoupling, whereas emission factors and energy intensity played a significant role in promoting strong decoupling.

Inferring from the empirical results, the following implications for decoupling CO_2_ emissions and economic growth are key for policymaking for the realisation of an emissions reduction rate of 15–45% by 2030 in Ghana [57]. The continual rise in CO_2_ emissions and economic growth implies that renewable energy technologies should be encouraged for production in both the services and industrial sectors. This is expected to result in sustained reductions in CO_2_ emissions while ensuring economic growth [17]. Moreover, policies on the reduction of CO_2_ emissions in Ghana should target the drivers of CO_2_ emissions, especially economic activities, emission factors, and population growth. Also, to help CO2 emissions grow without being tied to economic growth, current policies like the National Energy Efficiency Action Plan (NEEAP) [58] and the Green Ghana Programme [59] should be implemented more strongly. These policies will change the structure of the economy and the amount of energy it uses towards renewable sources, which will promote decoupling in Ghana. Future studies could disaggregate the economic sectors into mining and telecommunication, among others, to investigate other sectors that contribute to CO_2_ emissions if data become available on Ghana.

## Data Availability

The datasets generated and/or analysed during the current study are available from the corresponding author on reasonable request.

## Appendix

See Table 6.

**Table 6:** Drivers of changes in total CO_2_ emissions (annual time series in tCO_2_)

Period	Δ${C}_{f, }$	Δ${C}_{I}$	Δ${C}_{ES}$	Δ${C}_{EA }$	Δ${C}_{P}$	Δ${C}_{TOT}$
1990–1991	−243.999	−121.093	−494.659	552.3312	77.41967	−230
1991–1992	502.5896	−54.2878	−326.84	317.2574	81.28092	520
1992–1993	53.12818	−18.644	−938.976	924.9837	89.50817	110
1993–1994	230.4739	−6.49955	−942.038	895.5043	92.55899	270
1994–1995	183.0738	11.63641	−1367.78	1326.502	96.57132	250
1995–1996	349.0511	−82.775	−1355.84	1368.182	101.3821	380
1996–1997	121.1879	16.93179	−865.493	821.6116	105.7612	200
1997–1998	1652.228	148.9045	−1042.84	924.6073	127.098	1810
1998–1999	395.3289	−39.1398	−982.526	951.1135	155.2237	480
1999–2000	552.162	−2993.11	−108.42	1558.718	150.6493	−840
2000–2001	578.0858	−433.682	−1686.92	1866.234	146.2859	470
2001–2002	1417.524	−654.57	−1249.25	1529.961	166.3346	1210
2002–2003	291.1393	−1486.03	−1298.43	2024.494	178.8342	−290
2003–2004	−335.23	−735.795	−731.538	1121.825	170.7378	−510
2004–2005	631.5942	−891.529	−683.377	1171.671	171.6415	400
2005–2006	1118.995	71.23724	−4813.01	4835.997	196.7775	1410
2006–2007	844.6855	−827.291	−1281.2	1658.409	225.3961	620
2007–2008	−263.546	−1166.17	−1381.47	2094.187	227.001	−490
2008–2009	888.1742	731.4718	−2005.17	1540.533	234.9875	1390
2009–2010	547.871	642.991	−2399.92	2146.959	262.0963	1200
2010–2011	581.157	−534.826	−2048.64	2779.335	282.9757	1060
2011–2012	1279.786	−23.3113	−2286.31	2727.317	312.5225	2010
2012–2013	469.0758	−4275.07	−2809.57	7066.024	339.5448	790
2013–2014	−436.007	−301.766	−2580.11	2341.266	336.6142	−640
2014–2015	967.6981	−305.214	−2259.73	2022.676	334.5707	760
2015–2016	−811.23	−304.775	−2692.48	2460.162	328.3211	−1020
2016–2017	879.3075	−303.564	−2561.49	2335.052	320.6944	670
2017–2018	−13.9733	2.935355	21.07283	−18.9334	−3.03769	−11.9362
1990–2018	12,430.33	−13,933	−43,173	51,343.98	5309.752	11,978.06

*Source*: Authors’ construct

## Footnotes

1 An emissions factor is a representative figure that makes an effort to connect the amount of a pollutant released into the atmosphere with the activity that caused it. The weight of the pollutant divided by the volume, length, or duration of the activity that is emitting the pollutant is typically how these factors are expressed. High EF values correspond to high emissions (IPCC, 2019).
